# Electrospinning of Cyclodextrin–Oligolactide Derivatives

**DOI:** 10.3390/biom13020203

**Published:** 2023-01-19

**Authors:** Alena Opalkova Siskova, Liviu Sacarescu, Andrej Opalek, Jaroslav Mosnacek, Cristian Peptu

**Affiliations:** 1 Polymer Institute of Slovak Academy of Sciences, Dúbravská Cesta 9, 84541 Bratislava, Slovakia; 2 “Petru Poni” Institute of Macromolecular Chemistry, Aleea Grigore Gica Voda 41A, 700487 Iasi, Romania; 3 Institute of Material and Machine Mechanics of the Slovak Academy of Sciences, Dúbravská cesta 9, 84513 Bratislava, Slovakia

**Keywords:** cyclodextrin, cyclodextrin–oligolactide, electrospinning, polymer-free fibers, electrospun mat

## Abstract

The materials used for the preparation of electrospun mats exhibit a large variety. Among them, cyclodextrins (CDs) and their derivatives have received thorough attention. Herein, we focus on the preparation of electrospun fibers based on biodegradable cyclodextrin–oligolactide (CDLA) derivatives, which may be qualified as polymer-free cyclodextrin. CDLA was prepared by ring opening of L-lactide initiated by the β-cyclodextrin. A clear structural image of the high-purity CDLA product was proved by MALDI MS. Preparation of the electrospun mats was optimized by taking into consideration the electrospinning parameters such as applied voltage, needle-to-collector distance, flow rate, the concentration of cyclodextrin solutions, and solvent type. The obtained electrospun fibers were morphologically characterized by scanning electron microscopy (SEM), transmission electron microscopy (TEM), and small-angle X-ray scattering (SAXS). SEM allowed the optimization of the electrospinning process to obtain beadless fibers with submicronic diameters. Further analysis by TEM and SAXS revealed the inner structural features of the CDLA-based filaments. Our results showed that the high purity CDLA materials, structurally well-defined at the molecular level, are suitable for the preparation of electrospun mats by using dimethylformamide or a water/acetonitrile mixture as electrospinning solvents, similar to lower molecular weight commercial cyclodextrin derivatives.

## 1. Introduction

Electrospinning of various materials represents a relatively simple technique for producing continuous fibers with diameters ranging from micro- to nanodimensional domains, which can form mats with a high ratio of surface area to volume [1]. Therefore, electrospun fibers were considered appealing for a wide range of applications in the biomedical field such as drug delivery, scaffolds for tissue engineering, filtration or absorption of biological molecules, etc. [2,3,4]. Among the materials used to obtain electrospun fibers, polymers are attractive due to certain properties that ensure successful fiber formation, such as physical interaction between macromolecular chains, which leads to chain entanglement and fiber formation [5,6]. Although cyclodextrins (CDs), cyclic oligosaccharides, are not able to have chain entanglement interactions in the same way as polymers, they were also used for the preparation of electrospun fibers in combination with polymers [7]. The incorporation of CD into electrospun nanofibers can be achieved by electrospinning of CD/polymer mixtures, polymers with covalently attached CD (in-chain or side-chain) [7]. A special category of electrospun fibers may be obtained using polymer-free cyclodextrins (neat cyclodextrins or their derivatives), as recently reviewed [7,8]. The electrospinning of cyclodextrin derivatives, without using a carrier polymer matrix, was first reported by Celebioglu and Uyar [9]. The authors showed that bead-free nanofibers could be obtained by electrospinning of highly concentrated methyl-β-cyclodextrin (average substitution degree—1.8 methyl groups per glycosidic unit) solution in water and dimethylformamide. Later, electrospun fibers were also obtained from various CD derivatives such as hydroxypropyl-(α-, β-, or γ-)cyclodextrin [10,11], sulfobutyl ether-β-cyclodextrin [12], or peracetylated β-cyclodextrin [13]. These studies demonstrated that physical interactions through hydrogen bonding between CD molecules play a central role in the formation of the fibers. Later studies revealed that fiber formation in ESP of polymer-free CD systems depends on the concentration and, consequently, on the viscosity of the electrospun solution, leading to intermolecular interactions, similar to polymer chain entanglement, at specific concentration values (e.g., entanglement concentration of 58 wt% for HP-β-CD/N,N-dimethylformamide solution) [14].

Therefore, highly concentrated CD solutions are required for successful fiber formation and the low solubility of native cyclodextrins (especially β-CD, the most frequently employed in various applications) represents a limiting factor. Different combinations of CD derivatives and solvents have been employed for the preparation of CD-based electrospun fibers [8]. Such combinations are based on the solubility of a specific CD derivative in a certain solvent and the synthesis of CD derivatives with improved solubility may greatly expand the range of possibilities in this respect. Such approaches proved that electrospun fibers based on neat cyclodextrins can be prepared using 10% (*w*/*v*) NaOH solutions for α- and β-CD [15], water/DMSO mixtures (1/1 *v*/*v*) for γ-CD [16], or ionic liquids for β-CD [17]. Other studies on HP-β-CD revealed that the addition of salt may improve the ESP process given the low conductivity of cyclic carbohydrates [11,18].

The preparation of electrospun fibers based on CD molecules leads to materials with a high contact surface-to-volume ratio. Such materials inherit the CD capacity of physical inclusion inside their cavity and, therefore, can be used in various types of applications, e.g., encapsulation of active principles, removal of pollutants, odor masking, drug delivery, and others, as reviewed [8]. The addition of guest molecules in the electrospinning solutions of CD or CD derivatives allows the formation of bead-free fibers while physical inclusion complexes are concomitantly formed. Although the polymer-free CD electrospun fibers lack mechanical strength when compared with various CD–polymer combinations and fast leaching of fibers usually occurs, neat CD (or CD derivative) fibers raised a great interest regarding the design and development of drug delivery systems based on “fast-dissolving” electrospun nanofibers [19].

Cyclodextrin derivatives (commercial products) such as those used in the above-mentioned studies are produced through chemical reactions with a certain degree of complexity [20,21,22]. On the other hand, cyclodextrin derivatives with improved solubility in water or organic solvents can be produced through ring opening of various cyclic esters only in the presence of cyclodextrins, in bulk or solution conditions [23,24,25,26,27,28]. More specifically, homogeneous products, β-CD modified with oligolactide (CDLA), were obtained by solution polymerization of lactide only in the presence of β-CD and were structurally described as CD derivatives selectively grafted with short oligoester chains on the smaller rim of the CD [26,27]. Such derivatives are attractive for bio-related applications because they are biodegradable [24,29] and the reactants needed for their synthesis are available from renewable resources.

Herein, we investigate the amenability of CDLA [26,27] to the electrospinning processing method, taking into consideration that the CD derivatives must preserve the inclusion ability specific to CD, given further applications, and, as a consequence, one must consider a low amount of bonded polymers (short polymer chains grafted on CD). Usually, when CD star polymers are prepared, e.g., CDLA [30], the high ratio between polymer and CD components may prevent the further use of CD cavities for complexation because of the steric effects (e.g., pseudorotaxane formation). Our approach considers a cyclodextrin polymer-free system, according to general knowledge [7,8]. The effect of various processing parameters such as applied voltage, flow rate, needle-to-collector distance, and polymer solution concentration, as well as the type of solvent on the overall morphology of the fibrous mats, is investigated and discussed. Scanning electron microscopy (SEM) was used to assess the effect of the mentioned parameters while transmission electron microscopy (TEM) and small angle X-ray scattering (SAXS) provided insight into the fibers’ structural features.

## 2. Materials and Methods

### 2.1. Materials

β-cyclodextrin (β-CD) (Cyclolab, Hungary) was dried at 80 °C under vacuum and over P_2_O_5_ for 72 h and kept in a desiccator under Ar and over P_2_O_5_; ⳑ-lactide (ⳑ-LA) (Purac) was recrystallized twice from ethyl acetate, dried under vacuum and sublimated before use; N,N-dimethylformamide (DMF) used for synthesis was distilled under vacuum and dried over 3 Å molecular sieves for 72 h; acetonitrile (AcN), N,N-dimethylformamide (DMF), 99%, and N,N-dimethylacetamide (DMAc), 99.5% solvents used for electrospinning were HPLC gradient grade from Alfa Aesar (Kandel, Germany); freshly distilled water was also used for the electrospinning experiments. Classical 2 mL plastic syringes from Chirana, s.r.o. (Stará Turá, Slovakia) were used as polymer solution reservoirs; metal needles from B.Braun Slovakia (Bratislava, Slovakia) with dimensions 0.80 × 120 mm (21G×4¾″) and with flat tips were used for feeding the solutions to the electric field as well as for charging the solution during electrospinning. Voltage during electrospinning was driven by a high-voltage power supplier (Spellman SL-150W, Bochum, Germany). The solutions were fed by a single syringe pump model NE-1000 (New Era Pump Systems, Inc., Farmingdale, NY, USA). Aluminum foil was used as a static collector.

### 2.2. Methods

#### 2.2.1. Oligolactide-Modified β-Cyclodextrin Synthesis

The CDLA was prepared according to a previously reported procedure [27]. In a typical reaction, 2 g of β-CD and 2 g of ⳑ-LA were weighed together and added into a flame-dried flask containing a magnetic stir bar and 10 mL of dry DMF, under the protection of Ar flow. The flask, isolated with a rubber septum, was immersed in an oil bath over a heater with magnetic stirring and the temperature was brought to 85 °C. The ⳑ-LA monomer and the β-CD were completely dissolved in the reaction mixture after one hour. The heating was maintained for 48 h under continuous stirring. The reaction was stopped by simply removing the flask from the heating source and it was left to cool down before purification. The samples were purified by precipitation in cold diethyl ether and vacuum drying at 50 °C for 12 h to result in a fine white powder, with a yield of 85%.

^1^H-NMR (400.13 MHz, DMSO-d6, δ ppm): 5.94–5.70 (OH2, OH3), 5.5–5.42 (end-chain OH), 5.20–5.12 (CH, in-chain), 4.85 (H1), 4.65–4.18 (OH6, H6′, CH, end-chain), 3.90 (H5′), 3.64–3.37 (H3, H5, H6, H2, H4), 1.49–1.41 (CH3, in-chain), 1.3–1.28 (CH3, end-chain).

MALDI MS: Mn = 2250 g/mol, D = 1.039.

#### 2.2.2. Electrospinning Procedure

The CDLA solutions in different solvents (DMF, DMAc, water/AcN 1/1 *v*/*v* mixture—W/A) were prepared in four different concentrations: 150% (*w/v*) (4.5 g/3 mL), 160% (*w/v*) (4.8 g/3 mL), 170% (*w/v*) (5.1 g/3 mL), and 180% (*w/v*) (5.4 g/3 mL) and they were electrospun employing home-made syringe electrospinning apparatus in a horizontal arrangement. The positive voltage was controlled at 10, 15, 20, and 25 kV, respectively, to study the impact of applied voltage on the morphology and average diameter of fibers. The effects of two processing parameters, namely, flow rates of 0.1, 0.2, and 0.5 mL/h and needle–collector distances of 13, 15, and 17 cm, were studied.

#### 2.2.3. Structural Characterization of CDLA

*MALDI MS*: The raw samples withdrawn directly from the polymerization mixture were dissolved in a 1/1 W/A mixture to a concentration of 10 mg/mL. Afterward, the samples were mixed with a matrix solution (saturated solution of α-cyano-hydroxycinnamic acid in W/A mixture 1/1 *v/v*) in a ratio of 1/100 (*v/v*). One microliter of this mixture was deposited on a polished steel MALDI target. Mass spectra of polymers were measured on an UltrafleXtreme TOF instrument (Bruker, Bremen, Germany), equipped with a 355 nm smart beam-2 laser, capable of 1 kHz pulsing frequency. Mass spectrometer operation and spectra processing were performed by using FlexControl 3.3 software (Bruker, Bremen, Germany). The ionization laser power was adjusted to just above the threshold to produce charged species. More than 10,000 spectra were collected for each sample.

^1^H NMR CDLA: The proton NMR spectra were recorded on a Bruker Avance DRX 400 MHz spectrometer equipped with a 5 mm QNP direct detection probe and z-gradients. The spectra were recorded in DMSO-d6, at room temperature. The chemical shifts are reported as δ values (ppm) relative to the solvent residual peak. The obtained spectra coincided with the previously published ones [27].

#### 2.2.4. Morphology Studies

The morphology of the electrospun fibers was investigated by scanning electron microscopy (SEM), using a Jeol JSM 6610 (Jeol Ltd., Tokyo, Japan) with an accelerated voltage of 15kV. The samples were sputtered with a gold layer just before analysis. AzTec software Ver. 2.1. (Springfield, NJ, USA) was used for making figures and processing the results. The average diameter and dispersity of diameters were estimated on the basis of SEM images by using the freely accessible online software ImageJ Ver. 1.53e (LOCI, University of Wisconsin, Madison, WI, USA). The fiber diameter is expressed as average diameter ± standard deviation. One hundred fiber segments were analyzed randomly on 3 independently prepared samples to obtain a mean diameter for each type of nonwoven sample.

The measurements of the beads’ dimensions were carried out at the coarsest place on the individual beads. The average diameter was obtained from 50 measurements on 50 different beads.

Transmission electron microscopy (TEM) analysis was carried out with a Hitachi High-Tech HT7700 (Tokyo, Japan) instrument, operated in high contrast mode at 100 kV accelerating voltage. The samples were prepared by sandwiching the filaments between two uncovered 300 mesh copper grids (Ted Pella, Redding, CA, USA).

The proof of mechanical integrity was provided by using the Canon PowerShot SX130 camera (Tokyo, Japan).

#### 2.2.5. Small Angle X-ray Scattering

Small angle X-ray scattering (SAXS) experiments were performed using a three-pinhole collimation Bruker NanostarU (Karlsruhe, Germany) system equipped with a copper anode IμS microsource. The scattering intensity was recorded using a Vantec-2000 with a 68 μm resolution detector. The scattering intensity I(q) is plotted as a function of the momentum transfer vector q = 4πsinθ/λ, where λ is the wavelength of the X-rays (Cu Kα radiation, 1.54 Å), and θ is half of the scattering angle. The sample-to-detector distance was 107 cm, allowing measurements with q values between 0.008 Å-1 and 0.2 Å-1. The angular scale was calibrated by the scattering peaks of the silver behenate standard.

The samples for SAXS analysis were prepared by the detachment of a small part of the polymeric mat from the metal support and it was mounted on a dedicated holder using Kapton tape. The samples were measured under constant temperature, 25 °C for 20,000 s. The raw data were corrected for the transmission coefficient and the incoherent scattering due to the Kapton tape and the background was subtracted in the data analysis using the SAXS Bruker A integrated software V4.1.45. The data analysis was carried out using ATSAS 2.5.1 [31].

## 3. Results and Discussion

**Figure 1:**
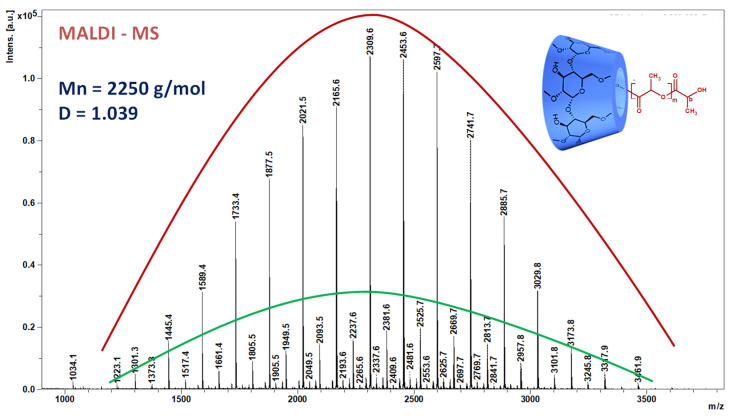
The MALDI MS spectrum of CDLA derivatives: the red line indicates the main series of peaks with an even number of lactate units and the green line indicates the secondary series of peaks with an odd number of lactate units.

Commercial cyclodextrin derivatives having well-defined structures and physical properties were extensively tested for polymer-free electrospinning [7,8]. The custom-prepared cyclodextrin–oligolactide (CDLA) derivatives employed in our study are described as cyclodextrins esterified at the primary OH groups with short oligolactide chains. They were prepared through ring-opening oligomerization (ROO) of ⳑ-lactide initiated by β-CD [27]. In this reaction, the presence of DMF solvent ensures an activation effect on the OH-initiating groups (in the given reaction conditions), thus, additional catalysts are unnecessary. Typically, the structure of the CDLA product may be proven by MALDI MS and MS/MS fragmentation studies (total number of lactate monomer units per CD molecule) and ^1^H and ^13^C NMR experiments (general structural sample description, substitution site, and average length of oligolactide branches) [27]. Herein, we present the characteristic MALDI MS spectrum of CDLA derivatives, shown in Figure 1, for product profiling purposes. The number of monomer units attached to a β-CD molecule ranges from 1 to 16 dilactate units (an *m/z* difference of 144 between two consecutive peaks is assigned to the dilactate unit). Any peak belonging to this series may be ascribed using the following formula: *m/z = 1134 (CD) + n* × *144 (dilactate) + 23 (Na)*. It may be observed that for any given peak of this series, the number of monolactate units (*n*) is even, which signifies that the actual monomer units are dilactates, also justified by the peak-to-peak mass difference, *Δm/z = 144 (2* × *72) Da*. Besides the main series of peaks, there may be observed a second series with lower intensity; these peaks correspond to CDLA species formed via transesterification reactions. This assumption is justified by the 72 Da difference from the main series corresponding to the monolactate unit. The *m/z* value of each member of this less significant series may also be obtained using the following formula: *m/z = 1134 (CD) + n* × *72 (monolactate) + 23 (Na)*. All the members of this series have an odd number of lactate units. The significant relative intensity difference between these two series proves that, in the ROO of ⳑ-LA initiated by β-CD, the transesterification side reactions have a low occurrence for the considered conditions.

Thus, based on the MS analysis of CDLA we may ascertain that the CD molecules are tethered with a calculated average of 15 lactate units, corresponding to an average molar mass, *Mn*, of 2250 g/mol with Đ = 1.039, however, with most of the population possessing an even number of lactate units.

### 3.1. Optimization of the ESP Process

The preparation of the electrospun mats based on the CDLA derivatives was first studied using DMF as a solvent, thus aiming to determine the influence of the electrospinning (ESP) parameters (Table 1).

The choice of DMF was based on the fact that CDLA derivatives are highly soluble, allowing facile preparation of highly concentrated solutions necessary for the ESP process. Moreover, DMF has been successfully used as a carrier solvent for previous ESP of CD derivatives [9].

Generally, viscosity and conductivity are perquisites in ESP of polymer-free cyclodextrins [8] and, given the low conductivity of cyclodextrins and their derivatives, the concentration, which influences the solution’s viscosity, appears to be the most important parameter. Previous studies have established a range of concentrations for successful electrospinning of polymer-free CD, between 150% and 180% (*w/v)*, for different combinations of CD derivatives and solvents [9,10,11,12,13,14,15,16,17,18,19]. Usually, the concentration values used for the ESP systems are provided in the weight of the substance per volume of solvent [9]. This way is preferred due to the simplicity of the preparation description, allowing the comparison of different solvent systems. Some other authors preferred the weight concentration [18] but both considered studies are in agreement that such ranges of concentration (approximately 60–80% weight or 150–170% weight per volume) are the best fit for ESP of CD derivatives.

The CDLA fibers were obtained herein by electrospinning of DMF solutions prepared by gently stirring with a magnetic stirrer at room temperature. The solutions with 150% (*w/v*), 160% (*w/v*), 170% (*w/v*), and 180% (*w/v*) concentrations were prepared to find appropriate solution concentration values for successful fiber formation.

The influence of electrospinning parameters on the formation and morphology of the fibers is presented in Table S1. The assessment of the impact of applied parameters on the ESP results was performed by the inspection of SEM micrographs (Figure S1). The aim was to find optimal conditions for obtaining suitable nanofibrous mats with potential biomedical applications. Thus, from the morphology point of view, an absence of beads on the resulting fibers was considered a qualifying criterion.

As may be observed from Table S1 and associated SEM images (Figure S1), the formation of the fibers via ESP of CDLA from DMF solutions is mainly influenced by the concentration and also by the other parameters, such as needle–collector distance (NCD), flow rate, and applied voltage. In principle, increasing the concentration leads to a transition from beads to beaded fibers and finally to bead-free fibers [32]. In this case, the final morphology and average fiber diameters and their dispersity could be tuned by playing with previously mentioned process parameters. 

Studies on hydroxypropyl-β-cyclodextrin (HP-β-CD) water solutions showed that an increased concentration, in the range of 60–80% wt, leads to an exponential increase in viscosity, associated with strong hydrogen bonding [18]. The hydrogen bonds’ implication in the electrospinning of CD solutions was probed by the introduction of urea as an agent for the disruption of hydrogen bonding.

Generally, polymer solutions submitted to ESP are characterized by an entanglement concentration, which is also valid for cyclodextrin solutions. The ESP process results in particle formation instead of fiber formation for solutions with a concentration value below the entanglement value. Once the entanglement concentration is reached the ESP process results in the formation of fibers. Viscosity–shearing rate dependence experiments on HP-β-CD/DMF solutions confirmed that the increase in concentration leads to hydrogen bond entanglements and fiber formation in ESP [14]. Moreover, the entanglement concentration may be calculated by measuring the dependence of the viscosity of the solutions on concentration. The entanglement concentration was about 58% wt for the tested cyclodextrin derivative–solvent combination, and SEM images showed that, above this concentration, the ESP process results in bead-free fibers.

Herein, for CDLA/DMF solutions, we found that for fixed ESP parameters, increasing only the solution concentration leads to almost bead-free fibers, at 160% (*w*/*v)* concentration (samples 6, 7, and 8 in Table S1). Considering the low CDLA conductivity, we assume that, similarly to other CD derivatives, hydrogen bonding between CD derivative molecules leads to increased viscosity and fiber formation [14,18]. To justify the formation of the electrospun fibers starting from the CDLA derivatives, one may argue that the oligoester chains grafted on the CD may sterically entangle, thus improving the overall electrospinnability. However, according to the structural characterization [27], the oligoester chains are rather short, thus preventing possible typical polymer entanglements. Moreover, experiments performed at concentrations lower than 150% (*w/v)*, in DMF, showed that the ESP process leads to the formation of particles (Figure S4).

### 3.2. Effect of the Solvent

Usually, the results of the electrospinning process depend largely on the solvent volatility and polymer (cyclodextrin derivatives herein)–solvent interactions, making the solvent choice critically important. The typical solvents used in ESP of cyclodextrin derivatives are ionic liquids, DMF, DMAc, and water, as reviewed [7,8]. One of the most important prerequisites for a good solvent in polymer-free cyclodextrin ESP is related to its capacity to dissolve a high amount of cyclodextrin derivative. Limited solubility prevents reaching an entanglement concentration generally above 58% wt or 150% (*w/v*). The employed CDLA derivatives are highly soluble in DMF or DMAc above the entanglement concentration, but in pure water, they form nanoaggregates and partially precipitate due to hydrophobic–hydrophilic interactions. Only the lower molecular weight fraction is water soluble, as shown for the derivatives modified with an average of six lactate units per CD molecule (Mn below 1700 g/mol) [24]. Liquid chromatography experiments showed that water/acetonitrile (W/A) blends are suitable to fully dissolve CDLA [25]. Therefore, taking into consideration CDLA solubility and solvent volatility, we chose for our study DMF, DMAc, and water/acetonitrile (W/A) (1/1 *v/v* mixture) as solvents (ESP conditions are given in Table 2). Parameters such as applied voltage, flow rate, and NCD were kept constant. The mentioned parameters were selected as the most convenient based on the results presented in the Supplementary Materials (Tables S1–S3).

**Figure 2:**
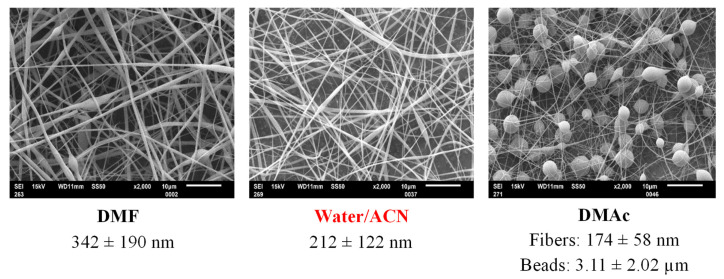
Representative SEM images obtained for the ESP of CDLA in different solvents, DMF, W/A, DMAc (ESP parameters: voltage—15 kV, working distance—13 cm, flow rate—0.1 mL/h, concentration—160% (*w/v*)).

The SEM micrographs of the electrospun mats presented in Figure 2 revealed that there are clear differences between employed CDLA/solvent combinations.

In the case of DMF, the fibers with larger diameters and the occasional presence of beads may be observed (Figure S1), while the DMAc preparation exhibits the predominant formation of beads and some thin fibers (Table S2 and Figure S2). The W/A mixture gave the best results at 160% (*w/v)* concentration, leading to CDLA fibers with the lowest diameter and optimal beadless morphological characteristics (Figure S3). The observed differences between the employed solvents demonstrate that entanglement phenomena leading to successful ESP processes are characteristic of specific CDLA–solvent combinations. For comparison, in the electrospinning experiments of hydroxypropyl-γ-cyclodextrin (HP-γ-CD) and hydroxypropyl-β-cyclodextrin (HP-β-CD), the ESP behavior of water, DMF, and DMAc solutions was relatively different, leading to the formation of beadless fibers, for different concentration ranges [10]. It can be concluded that the solvent type has a significant impact on the morphological properties of CD-based electrospun fibers, a fact which is in good agreement with other publications [9,10,11,12,13,14,15,16,17,18,19].

The oligolactide modification of β-CD improves the solubility of native β-CD in water because of the distortion of the stiff glycoside structure [24]. Thus, excellent improvement of β-CD water solubility was achieved by a CDLA product with relatively low molecular weight, approximately five lactate units per cyclodextrin molecule. In these conditions, a 70-fold increase in water solubility occurred for CDLA/amoxicillin complexes as compared with β-CD/amoxicillin (1.33 g/mL vs. 0.0018 g/mL, respectively) [24]. However, in our study, because of the higher amount of oligolactide (employed CDLA product has an average of 15 lactate units/CD), the water solubility decreased, and, as a consequence, water/organic solvent mixtures were required for complete solubility at the entanglement concentration.

**Figure 3:**
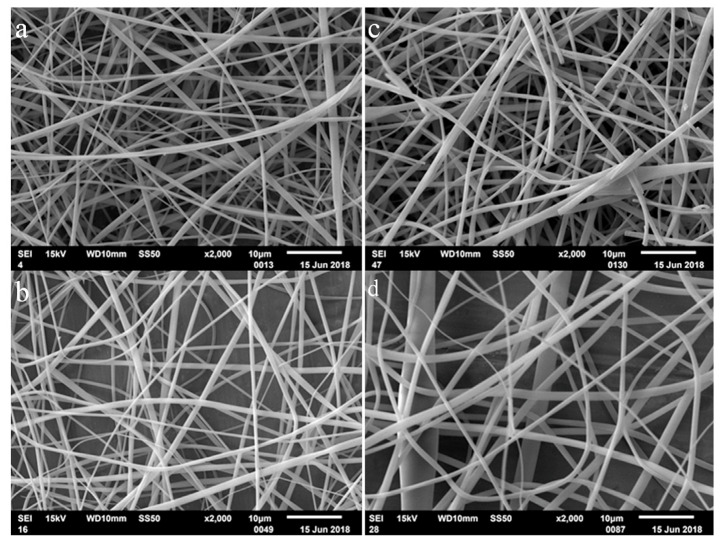
Comparison of SEM images of the ESP fibers obtained with increasing concentration in W/A mixture: 150% (*w/v*) (**a**), 160% (*w/v*) (**b**), 170% (*w/v*) (**c**), and 180% (*w/v*) (**d**) (0.1 mL/h, 25 kV, 13 cm NCD).

The W/A mixture, employed here the first time for cyclodextrin derivatives, demonstrated the best performance in the ESP preparation of beadless fibers, in the tested range of concentrations. The data presented in Table S3, together with the associated SEM images of the respective ESP preparations in the W/A mixture (Figure S3), show that a wider range of parameters may be used without the appearance of beads. Therefore, we may infer that the range of concentration values of CDLA in W/A solutions for successful ESP may exceed the maximal values tested herein. Nevertheless, in W/A solutions, CDLA fiber formation occurs for the entire range of tested concentrations (150% to 180% (*w/v*)) while, in the case of DMF, the concentration window seems to be narrow (above 160% (*w/v*)). At lower concentrations, e.g., 140% (*w/v*), the ESP of W/A solutions leads to particle formation instead of fibers (Figure S4). In the case of the W/A solvent mixture, there may be a tendency for fiber diameter growth with the increase in concentration values (Figure 3). Moreover, in rare cases (see Figure 3c), fiber fractures were observed. These fractures, caused by concentration changes during the electrospinning process, may be associated with the faster evaporation of acetonitrile.

The flow rate of the CDLA solution through the metallic needle tip determines the morphology of the electrospun materials. Uniform beadless electrospun nanofibers could be prepared via an optimal flow rate for a polymeric solution. Increasing the flow rate above the critical value may lead to the formation of beads [33] and an excess flow rate results in unequal mass dispersion [34,35]. The flow rate increase, together with the increase in concentration, triggers beads formation, as observed for the sample prepared at 0.5 mL/h and 180% (*w/v*) in the W/A mixture (sample 45 in Table S3 and Figure S3). Further, the beadless fibers are obtained by increasing the voltage (samples 46–48), thus showing the interdependence of the electrospinning parameters.

The observations in the present study (Table S3 and associated SEM images—Figure S3) confirm that the flow rate directly affects the fiber diameters and their dimensional dispersity. The W/A mixture leads to fibers for an increase in the ESP flow for all tested concentrations (between 150 and 180% *w/v*), up to 0.5 mL/h. However, increased flow rates lead to higher fiber diameter values, from 0.7 μm to 1.6 μm (samples 40 and 48 in Figure S3 and Table S3).

A series of experiments were carried out by setting the applied voltage at different values, 10, 15, 20, and 25 kV, respectively, while keeping constant the needle-to-collector distance, the concentration, and the flow rate, respectively. The preparations in the W/A mixture (Table S3 and Figure S3) revealed that, as a general tendency, the fiber diameter increases with the value of the applied voltage (e.g., at 180% (*w/v*), 0.1 mL/min the diameter increases from 0.4 to 0.7 μm for a voltage increasing from 10 to 25 kV). It has been shown that the applied voltage influences the force to pull a solution out from the needle in two ways [34]. First, increasing voltage leads to a larger fiber diameter due to an increase in the amount of solution mobilized out of the needle. Secondly, the applied voltage affects the charge density, thus the electrical force acts to elongate the jet during electrospinning, and, therefore, the fiber diameter increases.

We further tried to optimize the ESP process, using W/A as a solvent, by observing the effects of NCD on the morphology of the fibers. Thus, we noticed that NCD does not significantly affect fibrous morphology (Figures S5 and S6) and a fixed NCD of 13 cm was employed. However, when using DMF solvent, we noticed that a critical distance may be achieved at 15 cm. Additionally, similar results concerning the study of NCD showed that this parameter has no significant impact on the morphology and there is no change in the diameter of fibers in the distance interval between 5 and 35 cm [36]. However, according to others, increasing the distance up to the critical distance leads to a decrease in the diameters of forming fibers [37].

### 3.3. Mechanical Integrity

**Figure 4:**
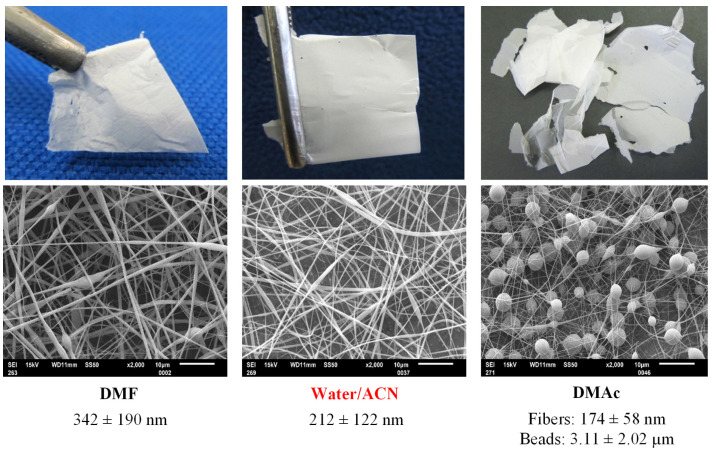
Photos of nanofibrous mats prepared by electrospinning, on the top, of 160% (*w/v*) solutions in DMF, in W/A, and DMAc (using 15 kV voltage, 0.1 mL/h of flow rate, and 15 cm) and, on the bottom, the associated SEM images.

The mechanical integrity of CDLA nanofibrous mats prepared from 160% (*w/v*) concentration solution in W/A, DMF, and DMAc (15 kV and 0.1 mL/h), was tested by visual examination of CDLA mats during simple manipulation (Figure 4). Compared to electrospun fibers from polymers, such as polyvinyl alcohol (PVA) [35], blends of poly(3-hydroxybutyrate-co-3-hydroxyvalerate)(PHBV)/polyethylene oxide (PEO) [38], or poly(L-lactic acid) [39], CDLA mats could be expected to be weaker and brittle because they are made from amorphous and relatively small molecules. However, as previously described, the mechanical integrity of mats based on β-CD [10] or even HP-β-CD with crystalline additives [40] was found to be satisfactory. Our observation also indicates that CDLA nanofibrous mats prepared from W/A and DMF can be handled as free-standing mats (Figure 4). On the contrary, the mats prepared from the DMAc solution were more brittle and difficult to handle because of their beaded structure. The prepared mats are fast spreading in a water environment, similar to other materials [19], and are expected to inherit the biodegradability as observed for other CLDA-based materials [29].

### 3.4. Structural Insights of CDLA Fibers

**Figure 5:**
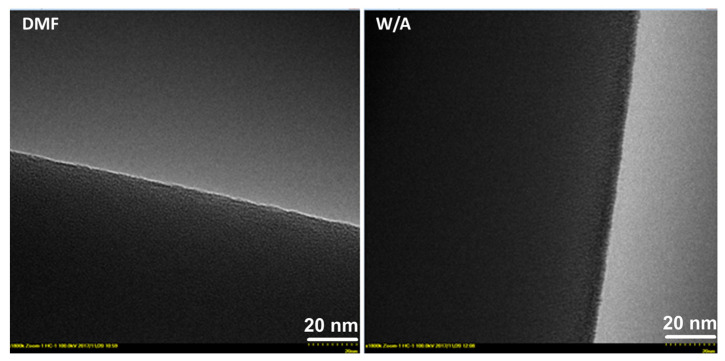
TEM micrographs at 1,800,000 × magnification showing electrospun fibers obtained in DMF and W/A at 160% (*w/v*).

The electrospun fibers, prepared from DMF and W/A, were also examined with high-resolution TEM (Figure 5). Both electrospinning systems presented a certain surface roughness but no porosity. With certain reserves, a granular morphology may also be observed. Similar TEM images were obtained by Manasco et al. [18] for HP-β-CD electrospun fibers. They assimilated the granular morphology with a possible presence of a self-assembled structure matching the individual dimensions of CD derivatives. Given the similitude between the fiber images obtained in our study and those mentioned above, we may infer a similar explanation for the observed granular formations. However, this unclear granular aspect could be caused by the lack of contrast in TEM, since both CD and lactide moieties composing the analyzed fibers have chemical structures based on carbon without any hard elements.

**Figure 6:**
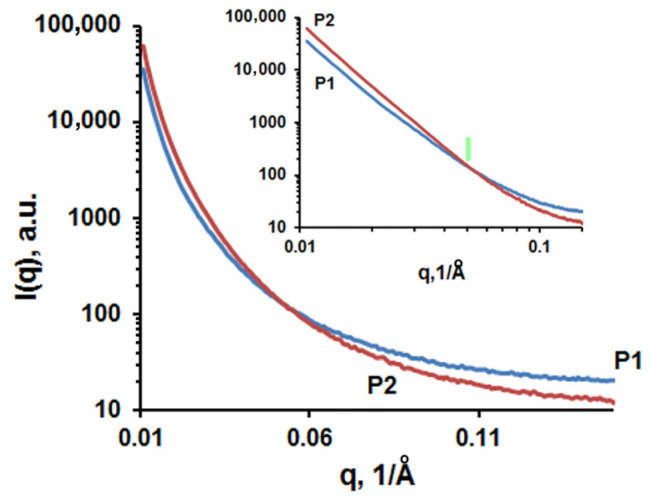
The scattering curves I(q) (log-norm plot) for samples obtained from W/A (P1—sample 13, Table S3) and DMF (P2—sample 7, Table S1), and inset—the logarithmic plot of the scattering curves. The green arrow indicates the end of the linear region corresponding to the same q value for both samples.

Therefore, we considered that it might be useful to further analyze the obtained fibers by SAXS. The scattering curves that describe the scattering intensity, I(q), versus the scattering vector, q, for the mats obtained from W/A (P1) and DMF (P2) are presented in Figure 6. Neither sample shows any specific features or peaks. This fact indicates the absence of an inter/intraphasic structural order within the SAXS dimensional range. Therefore, the interpretation of the SAXS data should take into account the TEM analysis of the fibers. First, one should observe that both samples have fibers’ diameters well above 100 nm. These scales are outside the upper dimensional limit of the SAXS measurements. On the other hand, at scales below 50–20 nm, the TEM micrographs show an unclear granular morphology.

At this point, the SAXS analysis can provide some useful information, knowing that this technique is based on scattering X-rays due to regions having different electron densities. In the studied case, these aspects correspond to regions located within the filament core, regions with dimensions below 100 nm. All this information corroborates to establish the context necessary for SAXS interpretation. Thus, the analyzed system is mainly a network of nanometric fibers. Within these fibers, one should consider the existence of domains having different electronic densities (scatterers). Such domains usually occur in materials processed by electrospinning. They are mostly pores and different materials as in composites or microphases. Re-considering the TEM micrographs, one should note that no pore structures could be evidenced within the fibers. In consequence, it is common sense to consider that the fibers contain two phases having different electron densities due to different packaging of the macromolecular structures or as a result of conformational effects. One of these phases represents the main CDLA matrix which is most likely homogeneous and the second one consists in domains that present a local heterogeneity induced by some structural disorder. The dimension of these heterogeneous domains could be estimated graphically using a Guinier plot [41]. According to this approach, at small q values, the scattering intensity follows a linear rule in terms of q:

$$I\left(q\right)=I\left(0\right)\;exp\left[-Rg^{2}q^{2}/3\right]$$

where Rg is gyration radius of the scatterers and I(0) is the scattering intensity for q → 0.

Then, the maximal average dimension of scatterers in real space could be estimated using the spherical body approximation as D = 2.58 × Rg. Thus, for the W/A sample, Rg = 21.7 nm and D_1max_ = 55.96 nm while for the DMF sample, Rg = 21.9 nm and D_2max_ = 56.5 nm.

According to these results, one can observe that the dimensions of the scatterers in both samples are similar. A possible reason is that the driving mechanism in the ESP processing method (hydrogen bonding entanglement) strongly affects the internal structure of the fibers, precluding the solvent influence. This observation is in agreement with the previous findings concerning the tendency of cyclodextrin derivative concentrated solutions to give local entanglements and to form self-aggregates [40]. Thus, light scattering measurements of highly concentrated HP-β-CD water solutions revealed the presence of aggregates with an average diameter of 10 nm. Such aggregates, estimated to contain around 30 cyclodextrin molecules for HP-β-CD, were hypothesized to form worm-like aggregates following the ESP process [18]. Given the higher molecular weight of CDLA molecules, we may assume a larger size of these aggregates composing the ESP fibers, thus explaining the Rg values around 20 nm.

A different approach to have additional information is to analyze the log–log plot of the I(q) function (Figure 6 inset). At first glance, one should observe the high similarity of the scattering curves for both samples. In addition, importantly, the linear region extends over several orders of magnitude of q. Such a profile suggests the fractal geometry of the scatterers.

For SAXS experiments, the intensity of the signal scattered by fractal objects could be described by:

$$I\left(q\right)=I_{0}q^{-a}$$

where I_0_ is a constant and α is the slope of the linear plot [42,43,44]. Depending on the value of α, one can determine the nature and dimension of the fractal. For the studied samples, the values of α indicate surface fractals with the following dimensions:

α = −3.52; Ds_1_ = 2.48 (from q_1_ = 10^−2^ to q_2_ = 5 × 10^−2^ Å^−1^), for CDLA fibers obtained in W/A;

α = −3.88; Ds_2_ = 2.12 (from q_1_ = 10^−2^ to q_2_ = 5 × 10^−2^ Å^−1^), for CDLA fibers obtained in DMF.

Thus, the fractal dimension Ds_2_ is close to the ideal value, 2, indicating a smooth perfect surface. Therefore, in the case of samples prepared in DMF, the matrix/scatter interface has a very low roughness. On the other hand, the sample obtained from W/A shows a higher roughness than the sample prepared in DMF. This difference could be the result of the scatterers differences between the studied samples induced by the different electrospinning conditions.

## 4. Conclusions

The electrospinning of CDLA derivatives resulted in the formation of mats composed of beadless fibers according to the employed process parameters, as observed by SEM analysis. Thus, DMF, DMAc, and a 1/1 *v/v* mixture of water and acetonitrile were tested. DMAc preparation led to beads on a string, ESP from DMF solutions led to fibers with a very low amount of beads, and the most appropriate solvent system for the preparation of CDLA fibers consists of a 1/1 *v/v* mixture of water and acetonitrile. The average diameter of the fibers ranged from 0.2 to 1.7 μm according to the tested ESP parameters (concentration, voltage, solvent flow, and needle-to-collector distance). The formation of the fibers in a similar manner as that observed for other CD derivatives (same range of entanglement concentrations) confirms the behavior of CDLA derivatives as polymer-free cyclodextrins, which form fibers due to hydrogen bonding entanglements. Moreover, high-resolution TEM analysis revealed that the CDLA fibers prepared in this study have the same morphological appearance, with a smooth surface, as compared with the fibers prepared from HP-β-CD, in other studies. The further employed analysis by SAXS revealed that the inner structure of the fibers is characterized by certain heterogeneity with fractal-shaped scatterers in the range of 55 nm. Current studies demonstrate the potential of CDLA derivatives to prepare electrospun fibers containing drug/CDLA complexes.

## Figures and Tables

**Table 1:** Process-related parameters applied for the electrospinning of CDLA solutions.

Electrospinning Parameters	Range
Solution-related parameters	
Concentration [% (*w/v*)]	150–180
Solvent	DMF, water/AcN, DMAc
Processing parameters	
Voltage [kV]	10–25
Feed rate [mL/h]	0.1–0.5
Needle-collector distance [cm]	13–17

**Table 2:** Electrospinning parameters—solvent effect.

Entry no.	Solution Concentration (*w/v*)	Solvent	Voltage [kV]	Flow Rate [mL/h]	NCD^1^ [cm]	Morphology	Fiber Diameter [nm]
1	160	DMF	15	0.1	13	Fibers ^2^	342 ± 190
2	160	H_2_O/ACN	15	0.1	13	Fibers	212 ± 122
3	160	DMAc	15	0.1	13	Beaded fibers	174 ± 58

^1^ NCD—Needle-to-collector distance. ^2^ Beads appeared rarely.

## Data Availability

Not applicable.

## Supplementary Materials

The following supporting information can be downloaded at: https://www.mdpi.com/article/10.3390/biom13020203/s1, Table S1. Electrospinning parameters of the electrospun CDLA fibers; Table S2. Electrospinning parameters—fibers prepared using N,N-dimethylacetamide (DMAc); Table S3. Electrospinning parameters—fibers prepared using water/acetonitrile; Figure S1. Representative SEM images of the electrospun CDLA nanofibrous mats obtained by application of different parameters during the electrospinning of CDLA solutions in DMF; SEM images correspond to the sample number as assigned in Table S1 (bar represents 10 μm); Figure S2. SEM images of the ESP fibers prepared using DMAc (numbers under images are associated with the preparation conditions given in Table S2); Figure S3. SEM images of the ESP fibers prepared using W/A mixture (numbers under each image are associated with the preparation conditions given in Table S3); Figure S4. SEM images of electrospun CDLA. The electrospinning conditions are the following: a—W/A solvent, 140% (*w/v*), 0.1 mL/h, 13 cm, 25 kV, b—W/A solvent, 130% (*w/v*), 0.1 mL/h, 13 cm, 25 kV, c—DMF solvent, 140% (*w/v*), 0.1 mL/h, 15 cm, 15 kV, d—DMF solvent, 130% (*w/v*), 0.1 mL/h, 15 cm, 15 kV; Figure S5. SEM images of the ESP fibers prepared using the W/A solvent, an applied voltage of 15kV, a flow rate of 0,1mL/h, and a concentration of 160% (*w/v*); Figure S6. SEM images of the ESP fibers prepared using the DMF solvent, an applied voltage of 15kV, a flow rate of 0,1mL/h, and a concentration of 160% (*w/v*).
