# Supplementary material for: Electrospinning of Cyclodextrin–Oligolactide Derivatives
Source: Biomolecules. 2023 Jan 19;13(2):203. doi: 10.3390/biom13020203 (PMC9953490; doi:10.3390/biom13020203)
Supplement: Supplementary file 1 [file biomolecules-13-00203-s001.zip › biomolecules-2139275-supplementary.pdf]

Supporting information

Electrospinning of cyclodextrin-oligolactide derivatives

Authors: Alena Opálková Šišková[1], Liviu Sacarescu[2], Andrej Opálek[3], Jaroslav Mosnáček[1], Cristian Peptu[1,2*]

Affiliations:

[1] Polymer Institute of the Slovak Academy of Sciences, Dúbravská Cesta 9, 84541 Bratislava, Slovakia;

[2] "Petru Poni" Institute of Macromolecular Chemistry, Aleea Grigore Gica Voda 41A, 700487 Iasi, Romania

[3] Institute of Material and Machine Mechanics of the Slovak Academy of Sciences, Dúbravská cesta 9, 84513 Bratislava, Slovakia

*Corresponding author – email: cristian_peptu@yahoo.com

Contents

Table S1. Electrospinning parameters of the electrospun CDLA fibers. – page S2

Figure S1. Representative SEM images of the electrospun CDLA nanofibrous mats obtained by application of different parameters during the electrospinning of CDLA solutions in DMF; SEM images correspond to the sample number as assigned in **Table S1** (bar represents 10 μm). – page S3

Table S2. Electrospinning parameters – fibers prepared using *N*,*N*-dimethylacetamide (DMAc). – page S4

Figure S2. SEM images of the ESP fibers prepared using DMAc (numbers under images are associated with the preparation conditions given in **Table S2**). – page S4

Table S3. Electrospinning parameters – fibers prepared using water/acetonitrile. – page S5

Figure S3. SEM images of the ESP fibers prepared using W/A mixture (numbers under each image are associated with the preparation conditions given in **Table S3**). – page S6

Figure S4. SEM images of electrospun CDLA. The electrospinning conditions are the following: a - W/A solvent, 140% w/v, 0.1 mL/h, 13 cm, 25 kV, b - W/A solvent, 130% w/v, 0.1 mL/h, 13 cm, 25 kV, c - DMF solvent, 140% w/v, 0.1 mL/h, 15 cm, 15 kV, d - DMF solvent, 130% w/v, 0.1 mL/h, 15 cm, 15 kV. – page S7

Figure S5. SEM images of the ESP fibers prepared using the W/A solvent, an applied voltage of 15kV, a flow rate of 0,1mL/h, and a concentration of 160% (w/v). – page S7

Figure S6. SEM images of the ESP fibers prepared using the DMF solvent, an applied voltage of 15kV, a flow rate of 0,1mL/h, and a concentration of 160% (w/v). – page S8

Table S1. Electrospinning parameters of the electrospun CDLA fibers.

Sample #	Solution concentration (w/v)	Solvent	Voltage [kV]	Flow rate [mL/h]	NCD[1] [cm]	Morphology	Fibers diameters [nm]
1	150%	DMF	10	0.5	13	Drops	-
2	150%	DMF	15	0.5	13	Beads [2]	243±80
3	150%	DMF	20	0.5	13	Beaded fibers [2]	238±76
4	150%	DMF	25	0.1	13	Fibers *	194±93
5	150%	DMF	25	0.1	15	Beaded fibers	254±109
6	160%	DMF	15	0.1	13	Fibers *	342±190
7	160%	DMF	15	0.1	15	Fibers *	272±132
8	160%	DMF	15	0.1	17	Fibers *	298±155
9	160%	DMF	15	0.2	13	Beaded fibers	202±91
10	160%	DMF	15	0.5	13	Beaded fibers	258±120
11	170%	DMF	15	0.5	13	Beads [2]	174±95
12	170%	DMF	25	0.2	13	Beaded fibers	200±84
13	170%	DMF	25	0.1	13	Beaded fibers	167±86
14	170%	DMF	25	0.1	15	Beaded fibers	206±123
15	180%	DMF	25	0.1	13	Fibers *	302±196
16	180%	DMF	25	0.1	15	Fibers *	261±162
17	180%	DMF	25	0.2	13	Beaded Fibers	212±148
18	180%	DMF	25	0.5	15	Beaded Fibers	277±144

Notes: [1] NCD–Needle-to-collector distance, * Beads appeared rarely, [2] Fibers rarely, # stands for no. and can be changed.

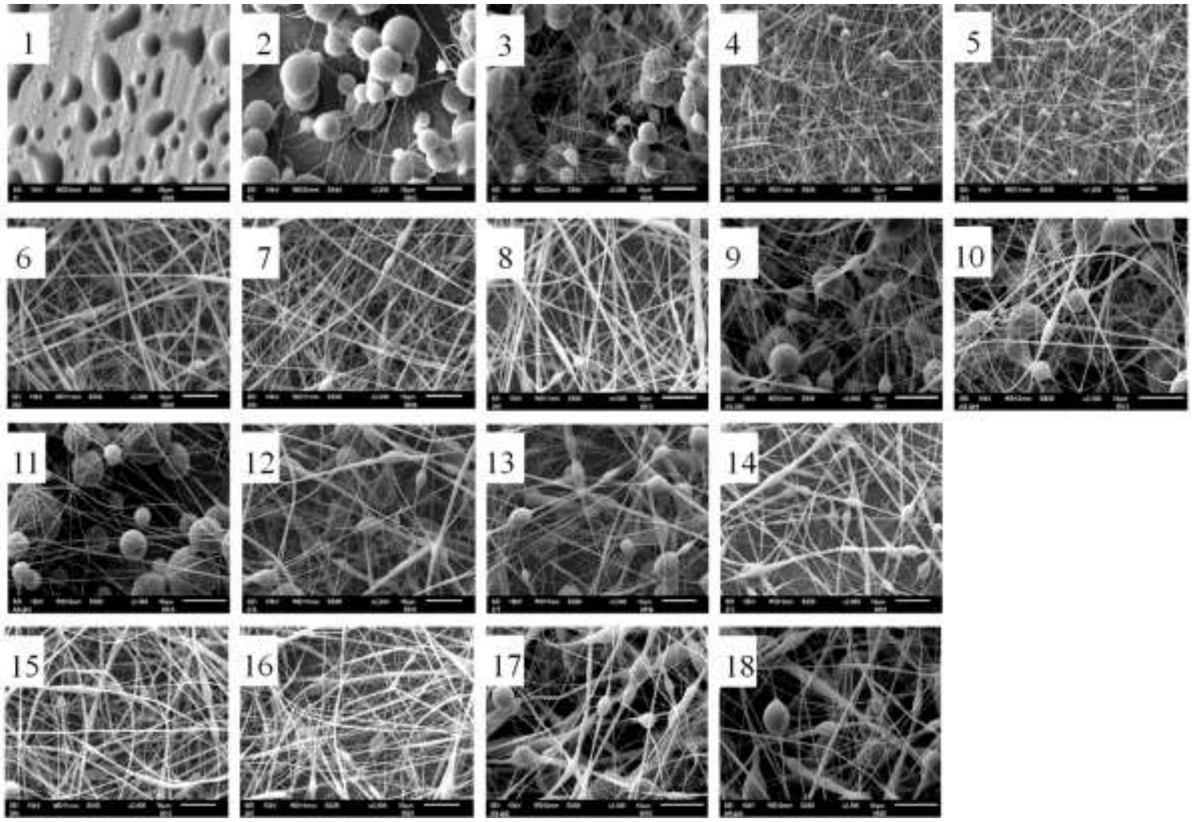

Figure S1. Representative SEM images of the electrospun CDLA nanofibrous mats obtained by application of different parameters during the electrospinning of CDLA solutions in DMF; numbers on the SEM images correspond to the sample numbers assigned in **Table S1** (bar represents 10 μm).

Table S2. Electrospinning parameters–fibers prepared using *N,N*-dimethylacetamide (DMAc).

Sample #	Solution concentration (w/v)	Solvent	Voltage [kV]	Flow rate [mL/h]	NCD[1] [cm]	Morphology	Fibers diameters [nm]
1	160%	DMAc	15	0.1	13	Beaded fibers	174±58
2	160%	DMAc	25	0.1	13	Beaded fibers	181±84

stands for no. and can be changed.

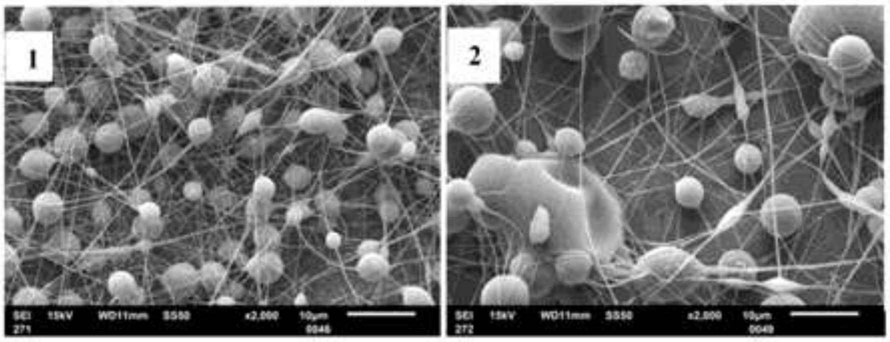

Figure S2. SEM images of the ESP fibers prepared using DMAc (numbers on the images are associated with the preparation conditions given in **Table S2**).

Table S3. Electrospinning parameters–fibers prepared using water/acetonitrile (NCD: 13 cm).

Sample no.	Solution concentration (w/w)	Voltage [kV]	Flow rate [mL/hour]	Morphology	Fibers diameters [nm]
1	150	10	0.1	Fibers	315 ± 304
2	150	15	0.1	Fibers	381 ± 241
3	150	20	0.1	Fibers	385 ± 288
4	150	25	0.1	Fibers	317 ± 186
5	150	10	0.2	Fibers	400 ± 169
6	150	15	0.2	Fibers	339 ± 163
7	150	20	0.2	Fibers	424 ± 238
8	150	25	0.2	Fibers	357 ± 188
9	150	10	0.5	Fibers	292 ± 120
10	150	15	0.5	Fibers	492 ± 733
11	150	20	0.5	Fibers	718 ± 495
12	150	25	0.5	Fibers	494 ± 528
13	160	10	0.1	Fibers	214 ± 88
14	160	15	0.1	Fibers	257 ± 155
15	160	20	0.1	Fibers	308 ± 222
16	160	25	0.1	Fibers	417 ± 218
17	160	10	0.2	Fibers	579 ± 1 454
18	160	15	0.2	Fibers	491 ± 375
19	160	20	0.2	Fibers	539 ± 299
20	160	25	0.2	Fibers	724 ± 530
21	160	10	0.5	Fibers	391 ± 447
22	160	15	0.5	Fibers	430 ± 239
23	160	20	0.5	Fibers	1 106 ± 813
24	160	25	0.5	Fibers	706 ± 566
25	170	10	0.1	Fibers	334 ± 375
26	170	15	0.1	Fibers	374 ± 350
27	170	20	0.1	Fibers	320 ± 136
28	170	25	0.1	Fibers	306 ± 175
29	170	10	0.2	Fibers	485 ± 599
30	170	15	0.2	Fibers	614 ± 233
31	170	20	0.2	Fibers	448 ± 303
32	170	25	0.2	Fibers	539 ± 453
33	170	10	0.5	Fibers	420 ± 398
34	170	15	0.5	Fibers	510 ± 396
35	170	20	0.5	Fibers	676 ± 512
36	170	25	0.5	Fibers	677 ± 276
37	180	10	0.1	Fibers	379 ± 202
38	180	15	0.1	Fibers	411 ± 309
39	180	20	0.1	Fibers	592 ± 567
40	180	25	0.1	Fibers	717 ± 567
41	180	10	0.2	Fibers	533 ± 199
42	180	15	0.2	Fibers	648 ± 356

43	180	20	0.2	Fibers	680 ± 500
44	180	25	0.2	Fibers	658 ± 326
45	180	10	0.5	Beaded	204 ± 117
46	180	15	0.5	Fibers	738 ± 756
47	180	20	0.5	Fibers	1 241 ± 472
48	180	25	0.5	Fibers	1 645 ± 842

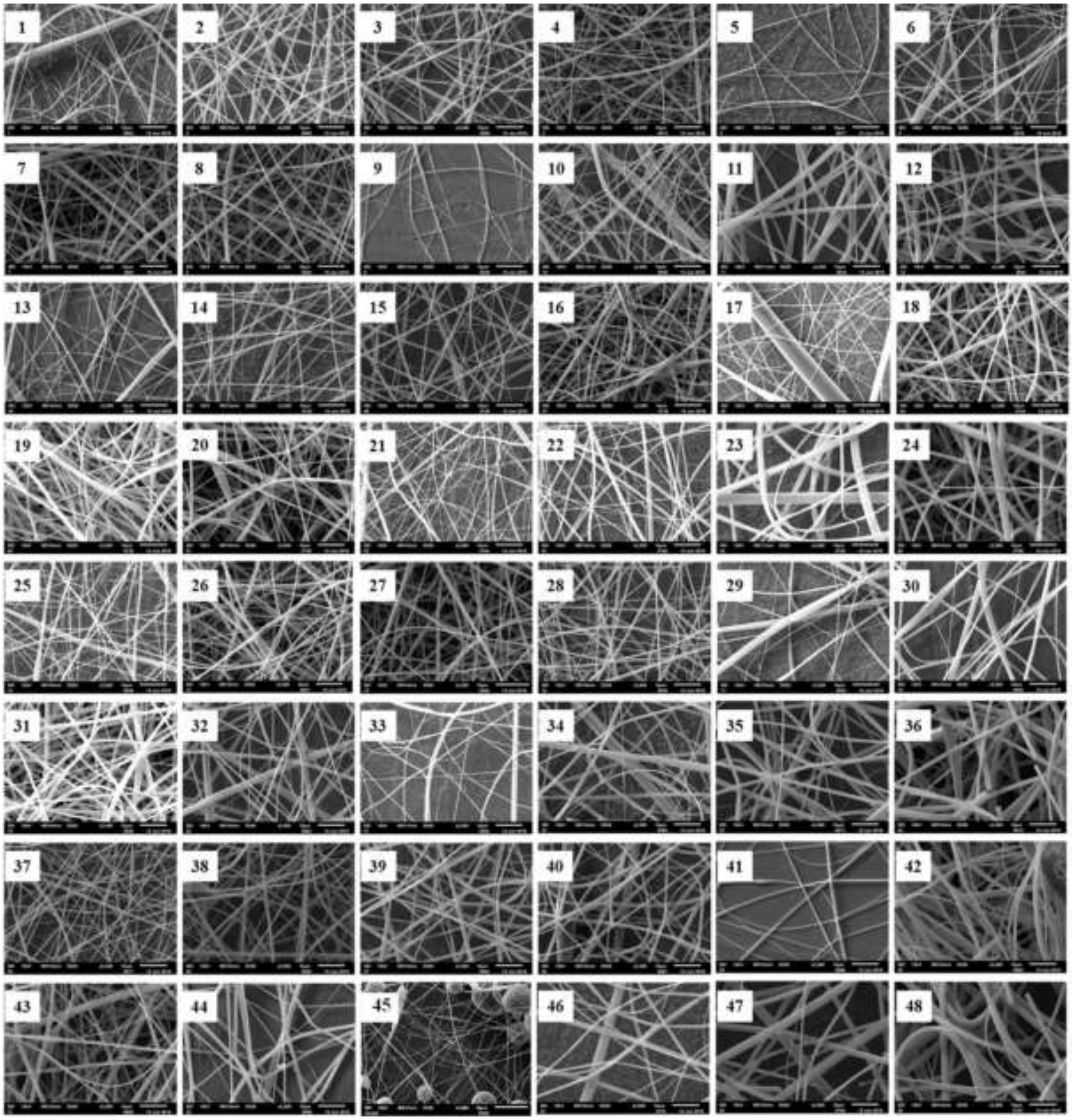

Figure S3. SEM images of the ESP fibers prepared using W/A mixture (numbers on each image are associated with the preparation conditions given in **Table S3**).

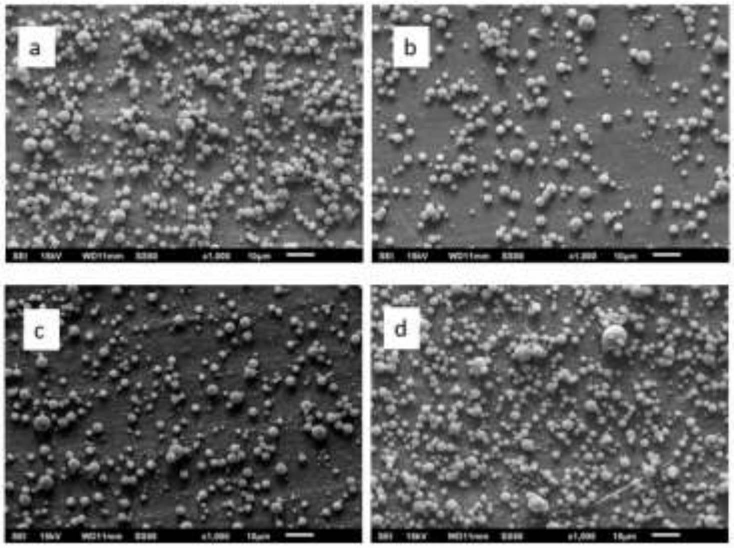

Figure S4. SEM images of electrospun CDLA. The electrospinning conditions are the following: a - W/A solvent, 140% w/v, 0.1 mL/h, 13 cm, 25 kV, b - W/A solvent, 130% w/v, 0.1 mL/h, 13 cm, 25 kV, c - DMF solvent, 140% w/v, 0.1 mL/h, 15 cm, 15 kV, d - DMF solvent, 130% w/v, 0.1 mL/h, 15 cm, 15 kV.

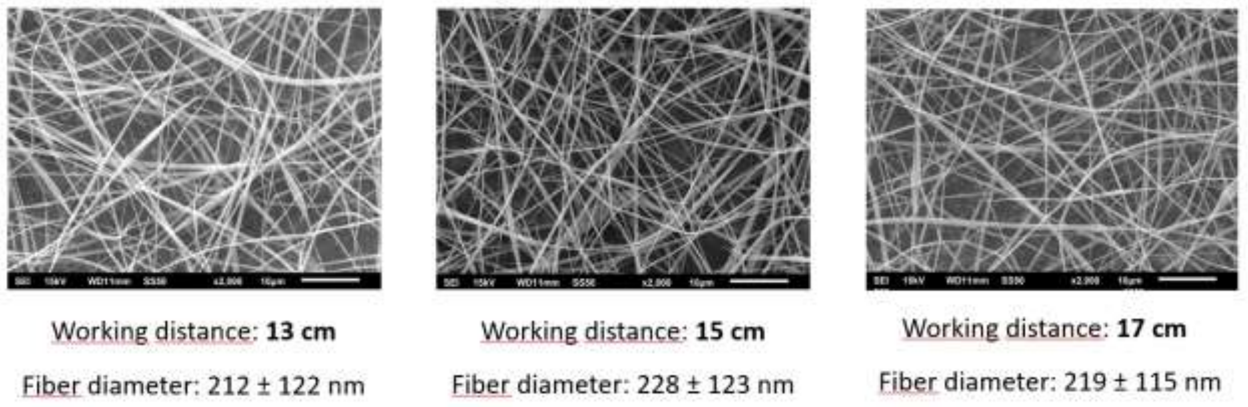

Figure S5. SEM images of the ESP fibers prepared using the W/A solvent, an applied voltage of 15kV, a flow rate of 0,1mL/h, and a concentration of 160% (w/v).

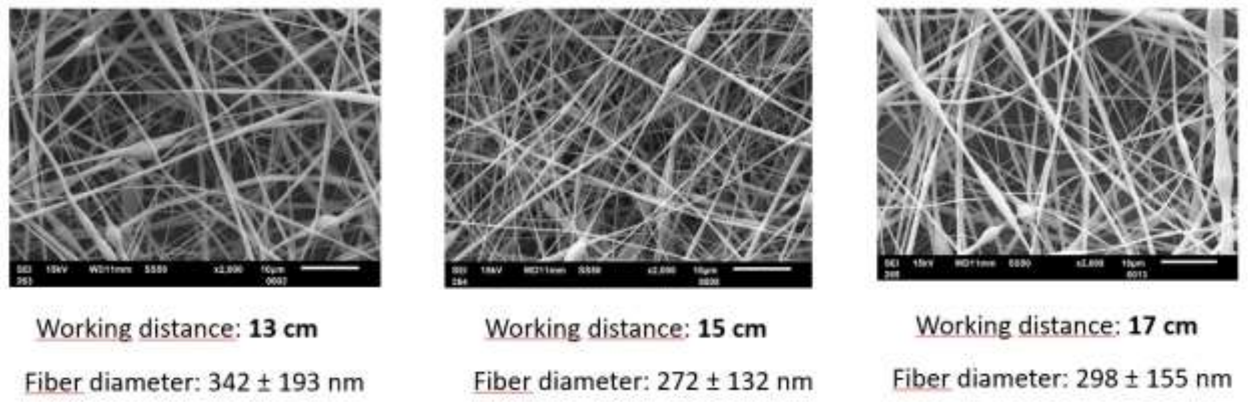

Figure S6. SEM images of the ESP fibers prepared using the DMF solvent, an applied voltage of 15kV, a flow rate of 0,1mL/h, and a concentration of 160% (w/v).
